# A multicentre case-control study of nonsteroidal anti-inflammatory drugs as a risk factor for severe sepsis and septic shock

**DOI:** 10.1186/cc7766

**Published:** 2009-03-30

**Authors:** Annick Legras, Bruno Giraudeau, Annie-Pierre Jonville-Bera, Christophe Camus, Bruno François, Isabelle Runge, Achille Kouatchet, Anne Veinstein, Jérome Tayoro, Daniel Villers, Elisabeth Autret-Leca

**Affiliations:** 1 Department of Intensive Care Unit, University Hospital of Tours, Boulevard Tonnellé, 37044 Tours, France; 2 INSERM CIC 202, François Rabelais University, Boulevard Tonnellé, 37044 Tours, France; 3 Department of Clinical Pharmacology and Regional Drug Monitoring Centre, University Hospital of Tours, Boulevard Tonnellé, 37044 Tours, France; 4 Department of Intensive Care Unit, University Hospital of Rennes, Rue Henri Le Guilloux, 35033 Rennes, France; 5 Department of Intensive Care Unit, CIC 0801, University Hospital of Limoges, Avenue Martin Luther King, 87000 Limoges, France; 6 Department of Intensive Care Unit, Regional Hospital of Orléans, Avenue de l'Hôpital, 45067 Orléans, France; 7 Department of Intensive Care Unit, University Hospital of Angers, Rue Larrey, 49033 Angers, France; 8 Department of Intensive Care Unit, University Hospital of Poitiers, Rue de la Milétrie, 86000 Poitiers, France; 9 Department of Intensive Care Unit, Hospital of Le Mans, Avenue Rubillard, 72037 Le Mans, France; 10 Department of Intensive Care Unit, University Hospital of Nantes, Place Alexis Ricordeau, 44093 Nantes, France; 11 François Rabelais University, Rue des Tanneurs, 37041 Tours, France

## Abstract

**Introduction:**

We aimed to establish whether the use of nonsteroidal anti-inflammatory drugs (NSAIDs) during evolving bacterial community-acquired infection in adults is associated with severe sepsis or septic shock.

**Methods:**

We conducted a multicentre case-control study in eight intensive care units. Cases were all adult patients admitted for severe sepsis or septic shock due to a bacterial community-acquired infection. Control individuals were patients hospitalized with a mild community-acquired infection. Each case was matched to one control for age, presence of diabetes and site of infection.

**Results:**

The main outcome measures were the proportions of cases and controls exposed to NSAIDs or aspirin during the period of observation. In all, 152 matched pairs were analyzed. The use of NSAIDs or aspirin during the observation period did not differ between cases and controls (27% versus 28; odds ratio = 0.93, 95% confidence interval [CI] = 0.52 to 1.64). If aspirin was not considered or if a distinction was made between acute and chronic drug treatment, there remained no difference between groups. However, the median time to prescription of effective antibiotic therapy was longer for NSAID users (6 days, 95% CI = 3 to 7 days) than for nonusers (3 days, 95% CI = 2 to 3 days; *P *= 0.02).

**Conclusions:**

In this study, the use of NSAIDs or aspirin during evolving bacterial infection was frequent and occurred in one-quarter of the patients with such infection. Although the use of NSAIDs by patients with severe sepsis or septic shock did not differ from their use by those with mild infection at the same infected site, we observed a longer median time to prescription of effective antibiotic therapy in NSAID users.

## Introduction

Nonsteroidal anti-inflammatory drugs (NSAIDs) and aspirin are widely used as antipyretic or analgesic drugs, even during evolving bacterial infections. Previous authors have described life-threatening infections associated with their use, mainly streptococcal infections and necrotizing fasciitis [1-4]. The involvement of NSAIDs in aggravation of bacterial infection is a matter of controversy [5,6]. A number of case reports concerning patients admitted to intensive care units (ICUs) have suggested that the use of NSAIDs increases the severity of bacterial infections and may lead to shock and multiple organ failure [7-10]. In the present study we investigated whether exposure to NSAIDs or aspirin affects the evolution of bacterial infection.

## Materials and methods

We carried out a multicentre case-control study in eight medical or polyvalent ICUs.

### Study population

All of the included patients were older than 15 years and had a bacterial community-acquired infection. All of the cases were patients admitted to an ICU with severe sepsis or septic shock [11,12]. Patients with one or more of the following were excluded: chronic kidney failure (creatinine clearance <30 ml/min), pregnancy, nosocomial infection, or congenital or acquired immunosuppression. Immunosuppression was defined as the presence of metastatic neoplasia, haemopathy, aplasia before the onset of sepsis, AIDS (independently of CD4^+ ^T-cell count) and chronic administration of immunosuppressive treatments, such as corticosteroids (equivalent of >30 days of prednisone at dosage >0.5 mg/kg per day), antineoplastic drugs or anti-tumour necrosis factor drugs.

Control individuals were patients admitted to hospital for mild bacterial community-acquired infection, defined as infection without any signs of severe sepsis or septic shock from the onset of the disease to their discharge from the hospital. Each case was matched to one control for age (± 10 years), presence of diabetes mellitus and site of infection (lung, urinary tract, skin and soft tissue, abdomen, genital tract, joints, heart, central nervous system or primary bloodstream). Diabetes was chosen for the matching process because it is a frequent chronic condition that increases the risk for severe infection. The site of infection was chosen for the matching process because the use of NSAIDs might differ depending on site of infection. The type of micro-organism was not considered in the matching process because bacterial documentation was not always available during sepsis.

This study was observational and did not require any deviation from routine practice. Our regional ethics review board approved the study. Informed consent was not required.

### Study design

**Figure 1:**
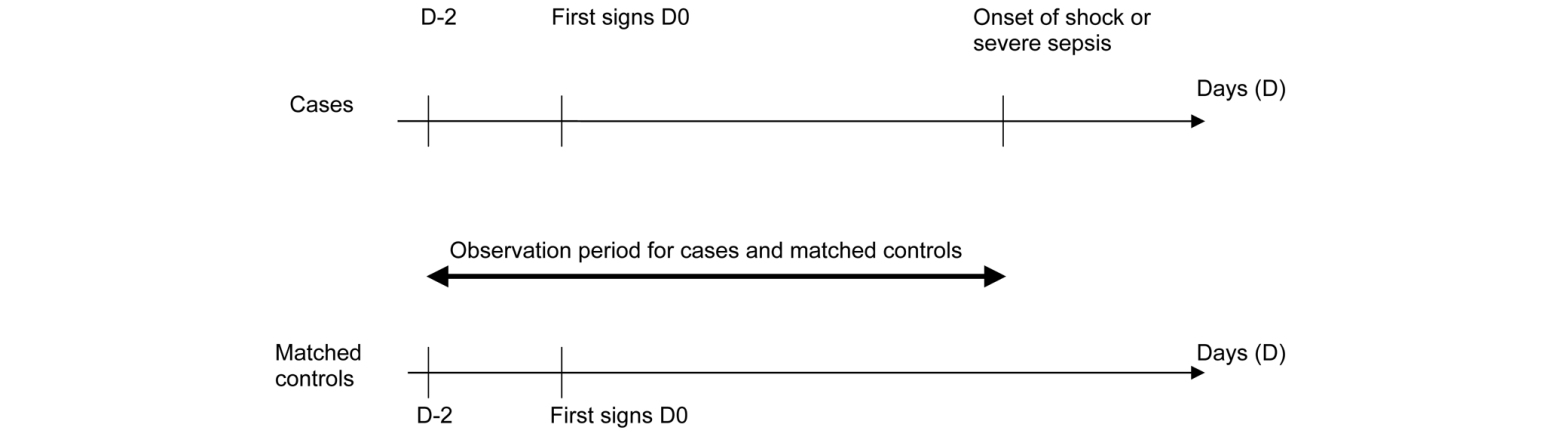
Observation period. The observation period for both cases and controls began 2 days before the onset of infection, and for the cases it lasted until the beginning of severe sepsis or septic shock.

For cases, the observation period began 2 days before the onset of infection, defined as the appearance of the first signs, and lasted until the beginning of severe sepsis or shock. Controls were observed for the same period (Figure 1). The duration of observation varied from one case-control pair to another, but it was identical for each case and matched control. NSAID use was quantified by careful listing of all of the drugs taken during the observation period, and standard interviews were conducted by physicians. An exhaustive list of all oral and parenteral NSAIDs (including their international nonproprietary name and brand name) was provided to each investigator. All NSAIDs and aspirin were considered. However, when aspirin was taken as an antiplatelet aggregant for the prevention of cardiovascular diseases (<350 mg/day), it was not taken into account. All types of oral and parenteral NSAID administration were considered (acute or chronic, prescribed or self-administered), whatever the duration or dosage. We defined acute administration of NSAIDs as their use for the observation period only, and chronic administration as their use for a chronic disease before that period.

Because most of the cases could not be interviewed on their admission to the ICUs, the recording of their medical history required the help of their relatives and general practitioner, as well as reference to previous prescriptions. Antibiotic therapy was studied and was considered effective if it exhibited appropriate *in vitro *activity and was appropriate for the pathogens isolated (or, in the case of culture-negative bacterial infection, for the suspected pathogens, based on international antibiotic therapy guidelines).

The main outcome measures of the study were the respective proportions of cases and controls who took prescribed or self-administered NSAIDs or aspirin during the observation period. We also compared, among the cases, the time from the first signs of infection to effective antibiotic therapy among NSAID users and nonusers.

### Statistical analysis

The study was planned as an investigation of matched pairs (one-to-one). Assuming an NSAID use rate of 20% among the controls and an odds ratio (OR) of two, we planned to recruit 150 pairs (alpha and beta risks were respectively fixed at 5% and 20%). ORs were estimated from discordant pairs, and exact 95% confidence intervals (CIs) were computed from the tail probabilities of the binomial distribution [13]. Adjustments for parameters whose distribution among cases and controls differed significantly were made within the framework of conditional logistic regression. Finally, the time to effective antibiotic therapy among cases was assessed using the Kaplan-Meier method, and NSAID users and nonusers were compared using the log-rank test [14]. Data were analyzed using SAS 9.1 software (SAS Institute Inc., Cary, NC, USA). Unless otherwise stated, values are expressed as mean ± standard deviation.

## Results

We recruited 152 cases from February 2004 to November 2005. They were matched to 152 controls. Table 1 shows the baseline characteristics of the cases and controls. Diabetes was present in 20 pairs (13%). The sites of infection were the lung (71 [47%]), urinary tract (30 [20%]), skin or soft tissues (16 [11%]), heart (11 [7%]), abdomen (10 [7%]), central nervous system (8 [5%]), joints (4 [3%]) and primary bloodstream (2 [1%]). A higher percentage of cases than controls had pre-existing neoplastic disease, chronic hepatopathy, were smokers or had a higher McCabe disease severity score. A higher percentage of controls had rheumatic disease. The median observation period for which total consumption of NSAIDs was estimated was 6 days (interquartile range = 5 days to 10 days; the minimum and maximum were 3 days and 32 days, respectively).

**Table 1:** Baseline characteristics of the 152 pairs of matched cases and controls

Characteristics	Cases (n = 152)	Controls (n = 152)	*P*
Male sex (*n *[%])	90 (59)	87 (57)	0.73
Age (years; mean ± SD)^a^	60 ± 15	61 ± 16	0.67
Body mass index (kg/m^2^; mean ± SD)	26 ± 6	26 ± 5	0.91
Current smoking (*n *[%])	57 (37)	38 (25)	0.02
Pre-existing disease (*n *[%])	54 (35)	62 (41)	0.34
Chronic respiratory failure (*n *[%])	22 (14)	15 (10)	0.22
Chronic heart failure (*n *[%])	12 (8)	14 (9)	0.68
Moderate chronic kidney failure (CrCl >30 ml/minute; *n *[%])	6 (4)	4 (3)	0.52
Chronic hepatopathy (*n *[%])	12 (8)	4 (3)	0.04
Pre-existing neoplastic disease (*n *[%])	12 (8)	2 (1)	0.01
Chronic rheumatic disease (*n *[%])	13 (9)	30 (20)	0.01
McCabe disease severity score (*n *[%])			
1	125 (82)	149 (98)	< 0.001
2	27 (18)	3 (2)	
3	0	0	

^a^Although age was a matching factor, a statistical test was performed because the matching window was fixed at ± 10 years. CrCl, creatinine clearance; SD, standard deviation.

On inclusion, the characteristics of the 152 cases included severe sepsis (34 [22%]) and septic shock (118 [78%]). The mean Simplified Acute Physiology Score II was 49 ± 20. The median length of stay in an ICU was 10 days (interquartile range = 4 days to 17 days). During hospitalization in an ICU, circulatory failure was present in 134 cases (88%), respiratory failure in 101 (67%), kidney failure in 79 (52%) and haematological failure in 37 (24%).

Bacteriological identification revealed the presence of one or more organisms in 123 out of 152 cases. The main organisms were *Streptococcus pneumoniae *(34 [28%]), *Escherichia coli *(29 [24%]), *Staphylococcus aureus *(19 [15%]) and *Streptococcus pyogenes *(7 [6%]). Antibiotic therapy before admission to an ICU proved ineffective in 74 cases (33%). Treatments included mechanical ventilation in 124 cases (82%), vasopressive drugs in 128 (84%), dialysis in 35 (23%), corticosteroids in 107 (70%) and drotrecogin alpha in 33 (22%). Surgery was performed to treat the origin of sepsis in 40 (26%) of the cases. The mortality rate in the ICUs among the cases was 24%.

All controls had a mild bacterial community-acquired infection. The median length of their hospital stay was 7 days (interquartile range = 5 days to 14 days). None of them developed severe sepsis or septic shock, none were admitted to an ICU and none died. Bacteriological identification revealed the presence of one or more micro-organisms in 75 out of 152 controls (49%). The main organisms were *Escherichia coli *(29 [39%]), *Staphylococcus aureus *(11 [15%]) and *Streptococcus pneumoniae *(7 [9%]). Only one *Streptococcus pyogenes *infection was identified.

The use of NSAIDs or aspirin during the observation period did not differ between cases and controls (27% versus 28; OR = 0.93, 95% CI = 0.52 to 1.64; *P *= 0.79; Table 2). If aspirin was not considered, there was still no difference, and no difference either when acute and chronic NSAID treatments were considered separately. If aspirin was not considered or if acute and chronic NSAID treatment were considered separately, there remained no difference between groups. Whether the duration of exposure taken into account was 1 day, more than 1 day or more than 2 days, or whether the end of the observation period was defined as the day of hospital admission rather than the beginning of severe sepsis or shock, NSAID or aspirin ingestion did not differ between cases and controls (data not shown).

**Table 2:** Comparison of NSAID and aspirin use by cases versus controls

Analysis		NSAID users (%)		OR (95% CI)	*P *value
				
		Cases	Controls		
Global (n = 152)^a^	NSAIDs and aspirin	27	28	0.93 (0.52 to 1.64)	0.79
	NSAIDs	24	21	1.18 (0.64 to 2.19)	0.56
	Chronic treatment	4	5	0.86 (0.24 to 2.98)	0.78
	Acute treatment	20	16	1.40 (0.69 to 2.92)	0.32
	Aspirin	5	10	0.47 (0.16 to 1.22)	0.09

Subgroup analysis	Diabetes (n = 20)^a^				
	NSAIDs	20	5	4.00 (0.40 to 196.99)	0.18
	Aspirin	10	5	2.00 (0.40 to 196.99)	0.56
	No diabetes (n = 132)^a^				
	NSAIDs	24	23	1.05 (0.55 to 2.00)	0.88
	Aspirin	4	11	0.36 (0.10 to 1.05)	0.04^b^
	Site of infection: lung (n = 71)^a^				
	NSAIDs	14	15	0.90 (0.32 to 2.46)	0.82
	Aspirin	7	11	0.63 (0.16 to 2.17)	0.40
	Site of infection: urinary tract (n = 30)^a^				
	NSAIDs	27	30	0.83 (0.02 to 3.28)	0.76
	Aspirin	3	7	0.50 (0.01 to 9.60)	0.56
	Site of infection: skin and soft tissue (n = 16)^a^				
	NSAIDs	31	31	1.00 (0.07 to 13.80)	1.00
	Aspirin	0	6	- -	-
	Site of infection: other (n = 35)^a^				
	NSAIDs	37	20	2.50 (0.72 to 10.92)	0.11
	Aspirin	3	11	0.25 (0.01 to 2.53)	0.18

^a^The *n *values indicate the number of pairs. ^b^The apparent discordance between the confidence interval (CI) and statistical test results is due to the use of different but asymptotically equivalent statistical methods. NSAID, nonsteroidal anti-inflammatory drug; OR, odds ratio.

Finally, there was still no difference between the two groups after adjustment for pre-existing diseases or for treatment centre (data not shown). Few diabetic patients were included in the study (only 20 pairs). There was no difference between their NSAID or aspirin consumption and that of the rest of the population studied. However, more nondiabetic controls than cases used aspirin (11% versus 4%; OR = 0.36; *P *= 0.04). For the three main sites of infection (lung, urinary tract, and skin or soft tissue), NSAID use varied depending on the site. Twice as many cases and controls with urinary tract or skin and soft tissue infections used NSAIDs compared with those who had lung infections. We did not observe any difference between cases and controls for any of the sites studied.

**Figure 2:**
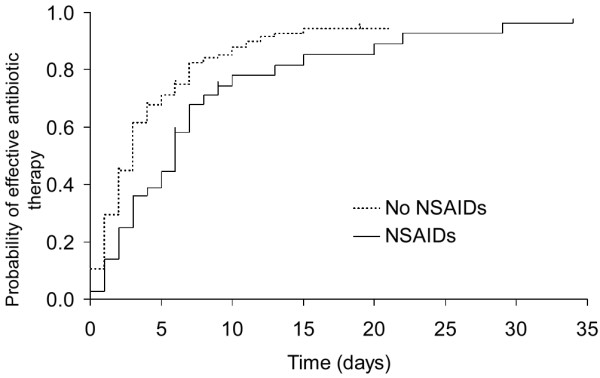
Time from the first signs of infection to effective antibiotic therapy. Shown is a comparison of the times from the first signs of infection to effective antibiotic therapy for cases; the compared groups were cases using nonsteroidal anti-inflammatory drugs (NSAIDs) and those not using these drugs. Log-rank test: *P *= 0.02.

Consequently, in the light of these findings, only the cases were studied. Among the cases, the time from the first signs to the prescription of effective antibiotic therapy was longer for NSAID users than for nonusers (median [95% CI]: 6 days, 3 days to 7 days for NSAID users versus 3 days, 2 days to 3 days for NSAID nonusers; *P *= 0.02; Figure 2). Among the cases, the mortality rate in NSAID users was 27% and that in nonusers was 23% (*P *= 0.58).

## Discussion

The findings presented here do not support the hypothesis that NSAID exposure during evolving bacterial infection is associated with an increased risk for severe sepsis or septic shock. However, in patients with severe sepsis or septic shock we observed that NSAID use is associated with a longer time from the first signs of infection to prescription of effective antibiotic therapy.

As stated in the Introduction (above), several case reports for patients admitted to ICUs [7-9] have suggested that NSAID treatment might increase the severity of infection and lead to shock and multiple organ failure. This is because life-threatening infections – mainly streptococcal, especially necrotizing fasciitis – have been described following NSAID use [3,5], as have infections with other organisms such as *Staphylococcus *spp. or Gram-negative bacilli, albeit less frequently [15]. However, unlike these case reports, case-control studies are designed to establish an association between an event and a risk factor and to quantify the risk involved. Most of the case-control studies relevant to the present investigation concerned the link between NSAID exposure of children with varicella and skin or soft tissue infections [16-19]. A significant association, which persisted after adjustment for age, sex and infection by micro-organisms (streptococci and other organisms), was found between ibuprofen use and necrotizing fasciitis [19]. This finding is particularly interesting, because the protopathic bias of the study was limited, as all cases had necrotizing fasciitis and all controls had severe post-varicella skin or soft tissue infections. As far as we know, our study is the first case-control investigation to focus on adults with community-acquired bacterial infections. Because the incidence of skin and soft tissue infections in ICUs is lower than that of lung or urinary tract infections [20], we included patients with many kinds of severe bacterial infections generally admitted to ICUs. The main site of infection was lung, for which fewer patients were given NSAIDs than for other infected sites, followed by urinary tract.

Several possible explanations can be suggested for our inability to find a link between NSAID use and increased risk for sepsis during bacterial infection. First, the sites of infection and micro-organisms that we identified, especially the low incidence of skin and soft tissue infections and consequently of streptococcal infections, differ from those most frequently identified in studies that found a link between NSAID and sepsis. We included various kinds of bacterial community-acquired infections, and among the 16 case/control pairs with skin and soft tissue infections *Streptococcus pyogenes *was identified in just seven cases and one control. Second, more microbiological documentation was available for cases than controls. However, this was not surprising, because the incidence of bacteraemia is usually higher in severe sepsis and septic shock, and because lung samples are more frequently available in patients with mechanical ventilation than in those without. The resulting high rate of undocumented infection in the control group may have biased the results of the study.

Finally, it is possible that we underestimated NSAID use among the cases. However, we assumed that more cases than controls took NSAIDs because the cases were more severely ill, and that NSAIDs were prescribed or self-administered to manage pain or fever. Such underestimation of NSAID use could have been due to the greater difficulty of assessing drug use in severely ill patients than in controls with mild infection, whose interviews provided more accurate information. Family questioning and analysis of initial prescriptions were mostly used in cases, and direct questioning in controls.

Other possible explanations for our negative findings are as follows: the study might have been underpowered (for instance, as a result of overestimation of NSAID use in cases); there may have been a sampling bias if the true population using NSAIDs was not representative of either the cases or the controls; and the patients with the most severe septic shock might have had no time to use NSAIDs.

Although the patients included in our study were suffering from ongoing infection, many had started taking NSAIDs before the beginning of effective antibiotic therapy. Among the cases, the median time from the first signs of infection to prescription of effective antibiotic therapy was twice as long for NSAID users as for nonusers (Figure 2). This was in agreement with the observations reported by Zerr and coworkers [19], who found a longer duration of secondary symptoms before hospitalization in NSAID-exposed than in NSAID-unexposed patients [19]. These findings suggest that NSAIDs probably delay the prescription of effective antibiotic therapy because they may mask the progression of disease by suppressing the inflammatory response induced by the infection [21,22]. This is a very important consideration, because delay in diagnosis and consequently in the administration of effective antibiotic therapy was recently shown to be one of the main risk factors for mortality [23].

The potentially harmful effect of NSAIDs may vary, depending on whether patients receive effective antibiotic therapy. Although this was not taken into account in our study, we observed higher mortality, albeit not significantly so, in NSAID-exposed patients. Certain other authors aimed to demonstrate, in contrast, that NSAIDs have a beneficial effect during sepsis, as observed in animals, and that inhibition of cyclo-oxygenase activity improves survival and reduces the physiological abnormalities caused by sepsis [22]. In adults given effective antibiotic therapy for sepsis, some investigators found no difference in clinical outcomes between NSAID users and nonusers, despite a decrease in prostacyclin metabolites in users [24-26]. However, the latter findings do not rule out the possibility that NSAIDs might be harmful in patients given ineffective antibiotic therapy. In any case, these drugs may predispose to severe bacterial infections because they inhibit leucocyte adherence, phagocytosis and bactericidal activity *in vitro *[22]. In addition, because NSAIDs have been found to increase inflammatory cytokine production in animal and human studies [24,27,28], and because the mortality rate for sepsis correlates with high interleukin-6 and tumour necrosis factor-α levels, the use of prostaglandin inhibitors in sepsis may be harmful. From this point of view, it might be useful to study infections that are more directly linked to the impairment of granulocyte function, such as fasciitis, extensive abscesses or collections of bacteria from different sites, rather than severe sepsis or septic shock, which are mainly the consequence of the systemic inflammatory reaction.

## Conclusions

The prescribed or self-administered use of NSAIDs is frequent during evolving bacterial infection, but in the present study it did not differ between patients with mild community acquired-infection and those with severe sepsis or septic shock. Our results therefore do not support the hypothesis that, during bacterial community-acquired infection, NSAIDs increase the risk for severe sepsis or septic shock. Nevertheless, NSAID use was associated with delayed prescription of effective antibiotic therapy. Further studies are needed to establish the effects of NSAIDs on patients whose antibiotic therapy is not effective, and whether NSAID use increases the morbidity of bacterial infections such as fasciitis or extensive abscesses, rather than the frequency of severe sepsis and septic shock.

## Key messages

• More than one-quarter of the patients who developed bacterial community-acquired infection were exposed to NSAIDs.

• For the patients with severe sepsis or septic shock who were given NSAIDs, the median interval between the first signs and the prescription of effective antibiotic therapy was longer than for those not given NSAIDs.

## Abbreviations

CI: confidence interval; ICU: intensive care unit; NSAID: nonsteroidal anti-inflammatory drug; OR: odds ratio.

## Competing interests

The authors declare that they have no competing interests.

## Authors' contributions

AL, BG, APJB and EAL participated in the design of the study and drafted the manuscript. AL, CC, BF, IR, AK, AV, JT and DV helped to collect study data. BG performed the statistical analysis. All authors read and approved the final manuscript.
